# Pulmonary Dysfunction Contributes to Nocturnal Hypoxemia Beyond Obstructive Sleep Apnea Severity in Primary Sjögren Syndrome

**DOI:** 10.3390/biomedicines14102152

**Published:** 2026-09-23

**Authors:** Emine Karabul, Erdal Mehmet Aksoy, Sermin Borekci, Serdar Ugurlu, Baran Balcan

**Affiliations:** 1 Department of Pulmonology, Koç University Hospital, 34010 Istanbul, Türkiye; karabullemine@gmail.com; 2 Department of Administration and Forensic Medicine, Koç University Hospital, 34010 Istanbul, Türkiye; eaksoy@kuh.ku.edu.tr; 3 Department of Pulmonology, Cerrahpasa Faculty of Medicine, Istanbul University, 34098 Istanbul, Türkiye; borekcisermin@gmail.com; 4 Department of Rheumatology, Cerrahpasa Faculty of Medicine, Istanbul University, 34098 Istanbul, Türkiye; serdalugurlu@gmail.com; 5 Department of Pulmonology, Faculty of Medicine, Koc University, 34010 Istanbul, Türkiye

**Keywords:** nocturnal hypoxemia, Sjögren syndrome, pulmonary dysfunction, obstructive sleep apnea

## Abstract

**Background/Objectives**: Pulmonary involvement is a common extraglandular manifestation of primary Sjögren syndrome (pSS) and may impair pulmonary gas exchange. Although obstructive sleep apnea (OSA) has been reported in pSS, the relationship between nocturnal hypoxemia and pulmonary dysfunction remains unclear. We investigated whether nocturnal oxygenation abnormalities in pSS are associated with pulmonary functional impairment beyond OSA severity. **Methods**: In this controlled cross-sectional study, 44 patients with pSS and 88 controls underwent overnight polysomnography. Pulmonary function testing, including diffusing capacity of the lung for carbon monoxide (DLCO), and thoracic computed tomography were available for all pSS patients. Associations between nocturnal hypoxemia parameters and pulmonary function indices were assessed using correlation analyses. Predictors of minimum nocturnal oxygen saturation were evaluated using multivariable linear regression adjusted for age, body mass index, and apnea–hypopnea index. **Results**: Sleep-disordered breathing severity and nocturnal oxygenation profiles were comparable between patients with pSS and controls, with the exception of a lower proportion of REM sleep in the pSS group. Within the pSS cohort, minimum nocturnal oxygen saturation correlated positively with DLCO, DLCO% predicted, DLCO/VA, and FVC% predicted. DLCO% predicted remained independently associated with minimum nocturnal oxygen saturation after adjustment for age, BMI, and AHI. However, this association was attenuated after additional adjustment for FVC, suggesting overlapping contributions of diffusion impairment and restrictive ventilatory dysfunction to nocturnal oxygenation. **Conclusions**: Although overall nocturnal oxygenation did not differ from that of matched controls, pulmonary functional impairment was associated with worse nocturnal oxygenation within the pSS cohort. These findings suggest that nocturnal hypoxemia in pSS reflects multiple interrelated aspects of pulmonary dysfunction rather than OSA severity alone. Assessment of pulmonary function, including both DLCO and FVC, may therefore provide clinically relevant information when evaluating nocturnal oxygenation abnormalities in patients with pSS.

## 1. Introduction

Primary Sjögren syndrome (pSS) is a chronic systemic autoimmune disease characterized by lymphocytic infiltration of exocrine glands and a wide spectrum of extraglandular manifestations. Among these manifestations, pulmonary involvement represents one of the most important causes of morbidity and mortality in affected patients [1,2].

Pulmonary manifestations of pSS include airway abnormalities, bronchiectasis, cystic lung disease, and interstitial lung disease (ILD). Importantly, pulmonary involvement may remain clinically silent for prolonged periods, and subclinical abnormalities are frequently detected on pulmonary function testing and thoracic imaging [1].

Recent systematic reviews and meta-analyses have demonstrated that ILD is present in approximately 20–25% of patients with pSS, with nonspecific interstitial pneumonia (NSIP) and usual interstitial pneumonia (UIP) being the most frequently observed radiological patterns. Reduced diffusing capacity of the lung for carbon monoxide (DLCO) is among the most common physiological abnormalities and is considered an important marker of pulmonary involvement [3,4]. Importantly, pulmonary involvement in pSS is heterogeneous and ranges from subclinical abnormalities detected only by pulmonary function testing to established interstitial lung disease visible on high-resolution computed tomography (HRCT). Because diffusion impairment may precede overt radiological abnormalities, physiological measurements such as DLCO may provide complementary information beyond structural imaging when evaluating pulmonary involvement. Whether these functional abnormalities contribute to sleep-related oxygenation independently of OSA severity remains largely unknown.

In addition to pulmonary manifestations, sleep disturbances are highly prevalent in pSS and contribute substantially to fatigue, impaired quality of life, and daytime dysfunction. Several polysomnographic studies have demonstrated a high prevalence of sleep-disordered breathing and obstructive sleep apnea (OSA) in patients with pSS [5,6].

However, previous investigations have primarily focused on the prevalence and severity of OSA as assessed by the apnea–hypopnea index (AHI), while largely overlooking pulmonary physiological factors that may influence nocturnal oxygenation [5]. Increasing evidence indicates that patients with similar AHI values may exhibit markedly different nocturnal oxygenation profiles depending on underlying pulmonary gas exchange, ventilation–perfusion matching, and respiratory reserve. Therefore, AHI alone may not fully capture the mechanisms contributing to nocturnal hypoxemia in patients with chronic pulmonary disorders, including connective tissue diseases [7,8]. In individuals with pulmonary parenchymal abnormalities and impaired gas exchange, nocturnal oxygenation may be influenced not only by upper airway obstruction but also by underlying pulmonary dysfunction. Consequently, patients with similar AHI values may demonstrate markedly different nocturnal oxygenation profiles despite comparable OSA severity [9].

Despite increasing recognition of both pulmonary involvement and sleep-disordered breathing in pSS, the relationship between nocturnal hypoxemia and pulmonary functional impairment remains poorly understood. In particular, whether nocturnal oxygenation abnormalities are associated with impaired DLCO and structural lung involvement independently of OSA severity has not been sufficiently investigated.

Therefore, the present study aimed to investigate whether nocturnal oxygenation abnormalities in patients with pSS are associated with pulmonary functional impairment beyond conventional measures of OSA severity. In addition to evaluating pulmonary function, we also explored whether structural pulmonary abnormalities detected on thoracic CT contributed to nocturnal oxygenation impairment. We hypothesized that impaired pulmonary physiology, particularly abnormalities in gas exchange, would be associated with worse nocturnal oxygenation independent of respiratory event frequency.

## 2. Materials and Methods

### 2.1. Study Design

This study was designed as a controlled cross-sectional observational study. Ethical approval was obtained from the Ethics Committee of Istanbul University-Cerrahpaşa, Cerrahpaşa Medical Faculty (IRB No. 604.01.01–45638). All procedures were conducted in accordance with the Declaration of Helsinki. Written informed consent was obtained from all participants prior to enrollment. Patients with primary Sjögren syndrome (pSS) were consecutively recruited from the Connective Tissue Diseases Outpatient Clinic of the Department of Chest Diseases between April 2019 and December 2020. The diagnosis of pSS was established by rheumatologists according to the contemporary classification criteria for primary Sjögren syndrome. Eligible participants were adults aged ≥18 years with a confirmed diagnosis of pSS who underwent overnight polysomnography (PSG), pulmonary function testing, and thoracic computed tomography (CT) as part of their routine clinical evaluation. Pulmonary function testing and thoracic CT were performed within 3 months of the polysomnographic evaluation. Exclusion criteria included the presence of another connective tissue disease, idiopathic interstitial pneumonia, sarcoidosis, amyloidosis, acromegaly, heart failure, active malignancy, pregnancy, insufficient sleep duration during PSG, or technically inadequate PSG recordings. The presence of exclusionary comorbidities was assessed through review of the participants’ medical history and available electronic medical records, including relevant clinical evaluations and imaging findings when available. A control group was retrospectively selected from individuals referred to the sleep disorders clinic during the same period who underwent PSG evaluation but had no diagnosis of pSS or other systemic connective tissue diseases. Control subjects were matched for age and sex whenever possible and were required to have no documented chronic pulmonary disease, normal chest imaging, and technically adequate PSG recordings. Pulmonary function testing was not routinely available in the control group.

### 2.2. Clinical Assessment

Demographic and clinical data were collected from medical records, including age, sex, smoking history, body mass index (BMI), neck circumference, symptoms of dry eye and dry mouth, snoring, witnessed apnea, and excessive daytime sleepiness. Epworth Sleepiness Scale (ESS) scores were recorded for all participants. For patients with pSS, available disease-specific characteristics included glandular manifestations, including dry eye and dry mouth symptoms, Schirmer test results and minor salivary gland biopsy findings, as well as serological characteristics including anti-Ro/SSA, anti-La/SSB, ANA and RF status. Pulmonary involvement was characterized using thoracic CT findings. Standardized measures of disease activity and severity, including ESSDAI and ESSPRI, were not routinely available because of the retrospective nature of the study.

### 2.3. Pulmonary Function Testing

Pulmonary function tests were performed according to international recommendations. A PFT device (Spirolab III; MIR, Waukesha, WI, USA) was used on each patient after being calibrated by the technician. Measurements included forced vital capacity (FVC), forced expiratory volume in one second (FEV1), FEV1/FVC ratio, diffusing capacity of the lung for carbon monoxide (DLCO), DLCO corrected for alveolar volume (DLCO/VA), and alveolar volume (VA). Both absolute values and percent predicted values were recorded when available. Hemoglobin-adjusted DLCO values were not routinely available in the institutional pulmonary function database. Therefore, conventional DLCO measurements obtained according to ATS/ERS recommendations were used throughout the analyses.

### 2.4. Thoracic Computed Tomography Evaluation

Thoracic CT examinations were reviewed for evidence of pulmonary involvement. Radiological findings including reticular infiltrates, ground-glass opacities, nodules, cysts, bronchiectasis, honeycombing, and interstitial lung disease patterns were recorded. Interstitial involvement burden and bronchiectatic involvement burden were quantified according to the number of affected pulmonary segments. For exploratory analyses, patients were also categorized according to the presence or absence of structural pulmonary involvement on thoracic CT whenever appropriate. Owing to the limited number of patients with individual radiological patterns such as NSIP or UIP, separate analyses for specific ILD subtypes were not prespecified.

### 2.5. Polysomnography

All participants underwent attended overnight polysomnography using the SOMNOscreen Plus system (SOMNOmedics, Randersacker, Germany). Sleep studies were scored independently by two experienced sleep physicians according to the American Academy of Sleep Medicine (AASM) scoring criteria. Recorded parameters included electroencephalography, electrooculography, electromyography, electrocardiography, nasal airflow, thoracoabdominal respiratory effort, body position, and pulse oximetry signals. Sleep architecture variables included total sleep time, sleep efficiency, sleep stage distribution, REM sleep percentage, and arousal index. Respiratory parameters included apnea–hypopnea index (AHI), REM-AHI, NREM-AHI, oxygen desaturation index (ODI), minimum oxygen saturation, and nocturnal oxygen desaturation events. Hypopnea was defined as a ≥30% reduction in airflow from pre-event baseline lasting ≥10 s and associated with a ≥3% decrease in oxygen saturation. Obstructive sleep apnea (OSA) was defined as an AHI ≥ 5 events/h. OSA severity was categorized as mild (AHI 5–14.9 events/h), moderate (AHI 15–29.9 events/h), or severe (AHI ≥ 30 events/h). Polysomnographic recordings were scored by experienced sleep physicians who were blinded to pulmonary function test results.

### 2.6. Definition of Nocturnal Hypoxemia

To characterize nocturnal oxygenation burden, several complementary hypoxemia metrics were evaluated, including minimum nocturnal oxygen saturation, total number of oxygen desaturation events, number of oxygen saturation drops below 90%, number of oxygen saturation drops below 80%, and the presence of nocturnal desaturation. Minimum nocturnal oxygen saturation was selected as the primary outcome variable for regression analyses. Minimum nocturnal oxygen saturation was selected as the primary outcome for regression analyses because it was consistently available in all participants and demonstrated the most consistent associations with pulmonary function parameters in preliminary analyses. Complementary oxygenation measures, including the total number of desaturation events and the number of events with SaO_2_ below 90% and 80%, were also evaluated to provide a broader characterization of nocturnal oxygenation.

### 2.7. Statistical Analysis

Continuous variables were expressed as mean ± standard deviation or median (interquartile range), as appropriate, whereas categorical variables were presented as frequencies and percentages. Between-group comparisons were performed using independent-samples *t*-tests or Mann–Whitney U tests for continuous variables and Pearson’s chi-square or Fisher’s exact tests for categorical variables. Correlation analyses between pulmonary function and nocturnal oxygenation parameters were performed in the entire pSS cohort (*n* = 44), irrespective of the presence or absence of structural pulmonary involvement on thoracic CT. Correlations between nocturnal hypoxemia parameters and pulmonary function variables (DLCO, DLCO%, DLCO/VA, and FVC%) were assessed using Spearman correlation analyses. Spearman correlation was used for bivariate analyses because several variables showed non-normal distributions and/or outlying values. For the linear regression analyses, model assumptions were assessed using residual diagnostics. Linearity, normality and homoscedasticity of residuals, multicollinearity, and influential observations were evaluated and found to be acceptable. To identify factors associated with nocturnal oxygenation impairment in patients with pSS, univariate linear regression analyses were initially performed using minimum nocturnal oxygen saturation as the dependent variable. Variables demonstrating a significance level of *p* < 0.10 in univariate analyses, together with clinically relevant covariates, were subsequently entered into multivariable linear regression models. Because pulmonary function parameters may reflect overlapping physiological domains, hierarchical multivariable regression models were constructed to evaluate the incremental contribution of pulmonary diffusion capacity and restrictive ventilatory impairment. Model 1 included age, BMI, and AHI. Model 2 additionally incorporated DLCO (% predicted), whereas Model 3 further included FVC (% predicted) to assess whether the association between DLCO and nocturnal oxygenation remained after accounting for restrictive lung physiology. For the exploratory thoracic CT analysis, patients with pSS were categorized according to the presence or absence of structural pulmonary involvement. Structural involvement was defined as the presence of at least one interstitial, cystic, or bronchiectatic abnormality on thoracic CT. Because several variables were non-normally distributed, comparisons between the two groups were performed using the Mann–Whitney U test. Statistical analyses were performed using IBM SPSS Statistics software, version 21, (IBM Corp., Armonk, NY, USA).

## 3. Results

### 3.1. Comparison of Sleep Characteristics and Nocturnal Oxygenation Between Patients with Primary Sjögren Syndrome and Controls

A total of 132 participants were included in the study, comprising 44 patients with primary Sjögren syndrome (PSS) and 88 control subjects. Baseline demographic and clinical characteristics are presented in Table 1. The two groups were generally comparable with respect to age (56.3 ± 13.2 vs. 54.0 ± 8.6 years, *p* = 0.172), body mass index (29.0 ± 4.6 vs. 28.5 ± 4.0 kg/m^2^, *p* = 0.422), Smoking exposure [0.0 (0.0–5.2) vs. 0.0 (0.0–15.0) pack-years, *p* = 0.191] and Epworth Sleepiness Scale scores [5.0 (3.0–7.2) vs. 5.0 (3.0–9.0), *p* = 0.579] were also comparable between groups. Neck circumference tended to be lower in patients with PSS than in controls [35.0 (33.0–36.2) vs. 36.0 (34.0–37.0) cm, *p* = 0.054]. As expected, the vast majority of participants in both groups were female (95.5% vs. 100.0%, *p* = 0.109). With regard to sleep architecture, the proportion of N3 sleep was similar between groups (20.8 ± 11.7% vs. 19.3 ± 8.4%, *p* = 0.690). However, patients with PSS exhibited a significantly lower percentage of REM sleep [12.0 (6.8–17.2)% vs. 15.5 (10.8–20.0)%, *p* = 0.025].

Sleep-disordered breathing parameters were largely comparable between groups. No significant differences were observed in overall AHI [12.0 (6.8–21.0) vs. 14.0 (6.8–22.2) events/h, *p* = 0.822], REM-AHI [26.0 (12.8–40.0) vs. 24.5 (10.0–38.5), *p* = 0.645], NREM-AHI [11.0 (5.0–18.0) vs. 10.0 (4.0–20.2), *p* = 0.950], supine AHI [14.0 (7.8–24.5) vs. 18.5 (7.0–36.0), *p* = 0.320], or non-supine AHI [6.0 (2.0–13.0) vs. 6.0 (2.0–15.0), *p* = 0.545]. Measures of nocturnal oxygenation were also broadly similar between groups. Minimum oxygen saturation during sleep was comparable in patients with PSS and controls [87.5 (81.0–90.0)% vs. 87.0 (83.0–89.0)%, *p* = 0.940], as was the number of oxygen desaturation events [73.5 (39.8–127.2) vs. 74.0 (39.0–139.5), *p* = 0.919]. Likewise, no significant differences were observed in the number of oxygen saturation events below 90% [4.0 (0.0–32.5) vs. 2.5 (1.0–25.0), *p* = 0.856] or below 80% [0.0 (0.0–0.0) vs. 0.0 (0.0–0.0), *p* = 0.672]. Overall, patients with primary Sjögren syndrome demonstrated a sleep-disordered breathing profile and nocturnal oxygenation burden comparable to those of the control group. The only significant difference identified was a lower proportion of REM sleep among patients with PSS.

Importantly, although no significant between-group differences were observed in nocturnal oxygenation parameters, substantial variability was present within the pSS cohort, providing the rationale for subsequent analyses examining the relationship between pulmonary function and nocturnal oxygenation.

### 3.2. Associations Between Pulmonary Function Parameters and Nocturnal Hypoxemia in Primary Sjögren Syndrome

In the entire pSS cohort (*n* = 44), irrespective of the presence or absence of structural pulmonary involvement on thoracic CT, lower pulmonary diffusing capacity was consistently associated with greater nocturnal oxygenation impairment (Table 2 and Figure 1). Minimum nocturnal oxygen saturation demonstrated moderate positive correlations with DLCO (r = 0.578, *p* < 0.001), DLCO% predicted (r = 0.556, *p* < 0.001), DLCO/VA (r = 0.300, *p* = 0.048), and FVC% predicted (r = 0.405, *p* = 0.006). Similarly, the number of oxygen saturation drops below 90% during sleep was inversely correlated with DLCO (r = −0.576, *p* < 0.001), DLCO% predicted (r = −0.541, *p* < 0.001), DLCO/VA (r = −0.297, *p* = 0.050), and FVC% predicted (r = −0.378, *p* = 0.011).

The total number of nocturnal desaturation events showed weaker but still significant inverse correlations with DLCO (r = −0.349, *p* = 0.020) and DLCO% predicted (r = −0.298, *p* = 0.049), whereas no significant associations were observed for DLCO/VA or FVC%. No statistically significant correlations were identified between pulmonary function parameters and the number of oxygen saturation events below 80%.

### 3.3. Predictors of Minimum Nocturnal Oxygen Saturation in Primary Sjögren Syndrome

To identify factors associated with nocturnal oxygenation impairment in patients with primary Sjögren syndrome, univariate and multivariable linear regression analyses were performed using minimum nocturnal oxygen saturation as the dependent variable (Table 3).

In univariate analyses, older age (β = −0.247, *p* < 0.001), higher BMI (β = −0.423, *p* = 0.033), and greater OSA severity as reflected by the apnea–hypopnea index (AHI) (β = −0.361, *p* < 0.001) were significantly associated with lower minimum nocturnal oxygen saturation. Among pulmonary function parameters, higher FVC (% predicted) (β = 0.137, *p* = 0.010), DLCO (% predicted) (β = 0.129, *p* < 0.001), and alveolar volume (VA) (β = 2.991, *p* < 0.001) were associated with higher minimum nocturnal oxygen saturation, whereas a higher FEV1/FVC ratio was weakly associated with lower minimum oxygen saturation (β = −0.265, *p* = 0.048). Neck circumference, smoking exposure, FEV1 (% predicted), DLCO/VA (% predicted), interstitial involvement burden, and bronchiectatic involvement burden were not significantly associated with minimum nocturnal oxygen saturation.

In the first multivariable model adjusted for age, BMI, and AHI, both age (β = −0.139, *p* = 0.014) and AHI (β = −0.270, *p* < 0.001) remained independently associated with lower minimum nocturnal oxygen saturation, whereas BMI was no longer significant (*p* = 0.225).

After inclusion of DLCO (% predicted) in Model 2, DLCO emerged as an independent predictor of minimum nocturnal oxygen saturation (β = 0.088, 95% CI 0.032 to 0.144, *p* = 0.003), while AHI remained significantly associated with nocturnal oxygenation impairment (β = −0.253, *p* < 0.001). Age and BMI were no longer statistically significant in this model.

In Model 3, which additionally included FVC (% predicted), both AHI (β = −0.238, *p* < 0.001) and FVC (% predicted) (β = 0.093, *p* = 0.017) remained independently associated with minimum nocturnal oxygen saturation. The association between DLCO (% predicted) and minimum nocturnal oxygen saturation was attenuated and no longer reached statistical significance (β = 0.053, *p* = 0.084).

Overall, the hierarchical regression analyses demonstrated that pulmonary function contributed additional information regarding nocturnal oxygenation beyond conventional OSA severity measures. DLCO (% predicted) remained independently associated with minimum nocturnal oxygen saturation after adjustment for age, BMI, and AHI. However, following additional adjustment for FVC, the magnitude of the DLCO association was attenuated and no longer reached statistical significance. These findings suggest that diffusion impairment and restrictive ventilatory dysfunction represent closely related physiological determinants of nocturnal oxygenation in patients with pSS rather than independent competing mechanisms.

### 3.4. Exploratory Analysis According to Thoracic CT Findings

As shown in Table 4, among the 44 patients with pSS, 23 had structural pulmonary involvement on thoracic CT, whereas 21 had no structural CT abnormalities. Patients with structural pulmonary involvement had significantly lower minimum nocturnal oxygen saturation than those without CT involvement [82.0% (interquartile range [IQR], 78.5–88.5) vs. 89.0% (IQR, 87.0–91.0), *p* = 0.002]. They also demonstrated a greater total number of nocturnal oxygen desaturation events [109.0 (IQR, 56.5–133.5) vs. 61.0 (IQR, 23.0–113.0), *p* = 0.041] and a higher number of oxygen saturation drops below 90% [20.0 (IQR, 2.0–68.0) vs. 0.0 (IQR, 0.0–5.0), *p* = 0.005]. The number of saturation drops below 80% was also higher in patients with structural CT involvement, although the difference did not reach statistical significance [0.0 (IQR, 0.0–0.5) vs. 0.0 (IQR, 0.0–0.0), *p* = 0.053]. Patients with structural pulmonary involvement additionally had higher AHI values [17.0 events/h (IQR, 9.5–22.5) vs. 9.0 events/h (IQR, 6.0–17.0), *p* = 0.047], lower FVC values [91.0% predicted (IQR, 88.5–104.5) vs. 110.0% predicted (IQR, 96.0–124.0), *p* = 0.010], and lower DLCO values [66.0% predicted (IQR, 52.0–71.0) vs. 91.0% predicted (IQR, 85.0–99.0), *p* < 0.001]. These exploratory findings suggest that structural pulmonary involvement on thoracic CT is associated with both impaired pulmonary function and a greater nocturnal oxygenation burden in patients with pSS. To further characterize the polysomnographic findings according to structural pulmonary involvement, exploratory comparisons with the control group were performed. Compared with controls, pSS patients with structural pulmonary involvement had lower minimum nocturnal oxygen saturation [82.0 (78.5–88.5)% vs. 87.0 (83.0–89.0)%, *p* = 0.033] and a lower proportion of REM sleep [12.0 (5.5–16.5)% vs. 15.5 (10.8–20.0)%, *p* = 0.028]. No significant differences were observed in the remaining evaluated polysomnographic parameters. In contrast, pSS patients without structural pulmonary involvement had higher minimum nocturnal oxygen saturation [89.0 (87.0–91.0)% vs. 87.0 (83.0–89.0)%, *p* = 0.017] and fewer oxygen saturation events below 90% [0.0 (0.0–5.0) vs. 2.5 (1.0–25.0), *p* = 0.019] than controls, while the remaining polysomnographic parameters were comparable. Detailed comparisons are provided in Supplementary Table S1.

## 4. Discussion

Our first important finding was that patients with pSS exhibited a sleep-disordered breathing profile largely comparable to that of control subjects. Neither OSA severity nor nocturnal oxygenation burden differed significantly between groups. These findings suggest that the presence of pSS alone may not necessarily translate into greater sleep-disordered breathing severity when compared with individuals referred for sleep evaluation who share similar demographic characteristics.

Previous studies investigating sleep-disordered breathing in pSS have reported conflicting results [5]. While some studies described an increased prevalence of OSA and excessive daytime sleepiness among patients with pSS, others failed to demonstrate major differences in objective polysomnographic respiratory parameters compared with control populations. Gudbjornsson et al. reported increased sleep complaints and fatigue in patients with pSS but found relatively modest alterations in objective sleep parameters, suggesting that subjective sleep impairment and respiratory sleep abnormalities may not always coexist in this population [10]. Similarly, several studies have reported a substantial burden of sleep-related symptoms, fatigue, and impaired sleep quality among patients with pSS, although objective polysomnographic abnormalities have been less consistently demonstrated [6]. Our results are generally consistent with these observations and suggest that pSS-related sleep complaints cannot be explained solely by greater OSA severity. The absence of significant differences between patients and controls should be interpreted cautiously. Because the control group consisted of subjects referred to a sleep clinic rather than healthy community controls, a relatively high prevalence of sleep-disordered breathing in the comparison group may have reduced between-group differences. Nevertheless, clinically relevant variability remained within the pSS cohort, allowing pulmonary function to emerge as an important determinant of nocturnal oxygenation.

An interesting observation in our study was the significantly lower proportion of REM sleep in patients with pSS. Although the underlying mechanisms remain uncertain, REM sleep alterations have been described in several chronic inflammatory and autoimmune diseases [11,12]. Experimental and clinical evidence suggests that inflammatory cytokines, chronic pain, fatigue, autonomic dysfunction, and persistent systemic immune activation may contribute to alterations in sleep architecture, particularly affecting REM sleep regulation [13]. Reduced REM sleep may contribute to impaired sleep quality, fatigue, and daytime dysfunction, symptoms that are frequently reported in patients with pSS [11,12]. Therefore, although respiratory sleep parameters were comparable between groups, the observed reduction in REM sleep may reflect disease-related alterations in sleep regulation that are independent of sleep-disordered breathing. However, this finding should be interpreted cautiously, as REM sleep may be influenced by several factors, including depression and antidepressant use. Because these variables were not systematically recorded in the present cohort, their potential contribution to the observed between-group difference could not be evaluated.

The principal finding of the present study was the strong association between pulmonary functional impairment and nocturnal hypoxemia in patients with pSS. Lower DLCO and DLCO% values were consistently associated with lower minimum nocturnal oxygen saturation and a greater burden of oxygen desaturation events during sleep. Similar associations were also observed for FVC%, whereas the strongest correlations were identified for DLCO-related parameters. These findings suggest that pulmonary physiological impairment contributes to nocturnal oxygenation abnormalities in pSS. However, reduced DLCO may reflect both parenchymal gas-exchange impairment and pulmonary vascular involvement; therefore, the present findings cannot distinguish their relative contributions. The attenuation of the DLCO association after adjustment for FVC suggests that these physiological measurements provide overlapping rather than competing information regarding pulmonary involvement. Therefore, our findings should not be interpreted as indicating that DLCO is the sole determinant of nocturnal oxygenation but rather as one component of a broader pulmonary physiological process. Consistent with this interpretation, patients with structural pulmonary abnormalities on thoracic CT exhibited lower minimum nocturnal oxygen saturation, more frequent nocturnal desaturation events, and significantly lower DLCO and FVC values than patients without CT abnormalities. These findings support the physiological relationship between structural lung involvement and impaired nocturnal oxygenation observed in our regression analyses.

Traditionally, the severity of sleep-disordered breathing has been evaluated using the apnea–hypopnea index (AHI). However, increasing evidence suggests that AHI alone incompletely reflects the physiological consequences of sleep-disordered breathing and may inadequately characterize the burden of nocturnal hypoxemia. Patients with similar AHI values may exhibit markedly different degrees of oxygen desaturation and associated clinical risk [7,8,9]. Consequently, several investigators have proposed that nocturnal hypoxemia and hypoxic burden may represent clinically meaningful physiological dimensions that extend beyond conventional respiratory event frequency measurements [7,8,9]. Our findings support this concept and suggest that pulmonary gas exchange abnormalities may represent an additional determinant of nocturnal oxygenation in pSS beyond upper airway obstruction alone.

The strong association observed between DLCO impairment and nocturnal hypoxemia in our cohort is biologically plausible. Reduced DLCO reflects impaired alveolar-capillary gas exchange and is one of the most sensitive physiological markers of pulmonary involvement in diffuse parenchymal lung diseases and connective tissue disease-associated interstitial lung disease [14,15]. During sleep, physiological reductions in ventilatory drive, changes in respiratory mechanics, and worsening ventilation-perfusion mismatch may further aggravate underlying gas exchange abnormalities, resulting in more pronounced nocturnal oxygen desaturation [16,17,18].

Our findings are also consistent with observations from interstitial lung disease (ILD) populations. Khor et al. demonstrated in a systematic review that nocturnal hypoxemia is frequently associated with impaired DLCO and pulmonary vascular abnormalities in patients with ILD [19]. Similarly, Troy et al. reported that nocturnal oxygen desaturation is associated with adverse clinical outcomes in ILD and may occur independently of conventional measures of respiratory event frequency [20]. Collectively, these findings support the concept that nocturnal oxygenation abnormalities may reflect pulmonary gas exchange impairment beyond upper airway obstruction alone.

Interestingly, DLCO demonstrated stronger associations with nocturnal oxygenation parameters than several conventional spirometric measurements. This observation may suggest that gas exchange abnormalities rather than ventilatory limitation represent the predominant mechanism underlying nocturnal oxygenation disturbances in pSS. From a clinical perspective, assessment of DLCO may therefore provide important information regarding sleep-related respiratory impairment that is not adequately captured by AHI alone.

The multivariable analyses provide additional insight into the mechanisms underlying nocturnal oxygenation abnormalities in pSS. While several demographic, sleep-related, and pulmonary function parameters were associated with minimum nocturnal oxygen saturation in univariate analyses, only a limited number of variables remained significant after adjustment for potential confounders. Most notably, DLCO (% predicted) remained independently associated with minimum nocturnal oxygen saturation after adjustment for age, BMI, and OSA severity, indicating that the relationship between pulmonary function impairment and nocturnal oxygenation cannot be explained solely by differences in body habitus or sleep-disordered breathing severity.

An important observation was that the inclusion of DLCO significantly improved the explanatory value of the regression model. This finding suggests that pulmonary gas transfer abnormalities contribute independently to nocturnal oxygenation and provide information beyond conventional OSA metrics. Similar observations have been reported in interstitial lung disease populations, where DLCO has consistently emerged as a sensitive physiological marker of gas exchange impairment and disease severity [14,21].

Interestingly, the independent effect of DLCO was attenuated after the addition of FVC to the final model. Although this finding may partly reflect collinearity between pulmonary function parameters, it may also suggest that nocturnal oxygenation abnormalities in pSS are influenced by multiple interrelated aspects of pulmonary involvement rather than a single physiological abnormality. From a pathophysiological perspective, both impaired gas transfer capacity and reduced lung volumes may contribute to nocturnal oxygen desaturation through complementary mechanisms [14].

Another notable finding was the absence of independent associations between nocturnal oxygenation and radiological extent of interstitial or bronchiectatic involvement. This observation may indicate that physiological measurements are more sensitive than structural imaging in identifying pulmonary abnormalities relevant to nocturnal gas exchange. Previous studies in connective tissue disease-associated interstitial lung disease have similarly demonstrated that physiological impairment may not always correlate closely with radiological disease extent, particularly in early or mild disease stages [21,22].

Taken together, these findings suggest that pulmonary function testing may provide clinically useful information regarding nocturnal respiratory impairment in patients with pSS. DLCO remained independently associated with minimum nocturnal oxygen saturation after adjustment for age, BMI, and AHI; however, this association was attenuated after FVC was incorporated into the final regression model. These findings suggest that impaired gas exchange and restrictive ventilatory dysfunction may contribute jointly to nocturnal oxygenation in patients with pSS, rather than DLCO representing an isolated independent determinant.

### Limitations

Several limitations of the present study should be acknowledged. First, the cross-sectional design precludes any conclusions regarding causal relationships between pulmonary function impairment and nocturnal hypoxemia. Second, the study was conducted at a single tertiary referral center and included a relatively limited number of patients with primary Sjögren syndrome, which may have reduced the statistical power to detect weaker associations and may limit the generalizability of the findings. Third, nocturnal oxygenation was assessed using conventional oximetry-derived parameters, including minimum oxygen saturation and desaturation event counts. More comprehensive measures of nocturnal hypoxemia burden, including mean nocturnal oxygen saturation, cumulative time spent below 90% oxygen saturation (T90), and hypoxic burden metrics, were not available in the archived PSG dataset. Future studies incorporating these measures may provide a more detailed characterization of sleep-related oxygenation abnormalities in pSS. Fourth, radiological pulmonary involvement was evaluated using the presence and extent of CT abnormalities rather than standardized quantitative imaging scores. Consequently, subtle associations between structural lung abnormalities and nocturnal oxygenation may have been underestimated. In addition, systematic echocardiographic data and CT-based pulmonary vascular measurements were unavailable; therefore, underlying pulmonary hypertension could not be specifically evaluated or excluded as a contributor to reduced DLCO and nocturnal oxygenation abnormalities. Pulmonary function testing was not systematically available in the control group; therefore, subclinical or previously undiagnosed airflow obstruction, particularly among participants with a smoking history, could not be definitively excluded despite normal chest imaging. Comprehensive data on systemic manifestations and standardized measures of pSS disease activity and severity, including ESSDAI and ESSPRI, were not available. Consequently, patients could not be reliably stratified into glandular-only and systemic disease phenotypes, and the potential influence of overall pSS disease severity and systemic involvement on sleep characteristics could not be evaluated. Future prospective studies incorporating standardized measures of disease activity and systemic involvement are warranted to address this relationship. Finally, because the study population consisted predominantly of women and included patients referred for sleep evaluation, the findings may not be fully representative of the broader pSS population. Additionally, Pulmonary function testing was unavailable in the control group; therefore, similar analyses could not be performed outside the pSS cohort. Hemoglobin-corrected DLCO measurements were not routinely available, and standardized disease activity indices such as ESSDAI were not consistently recorded because of the retrospective design.

## 5. Conclusions

Patients with primary Sjögren syndrome exhibited a sleep-disordered breathing profile and overall nocturnal oxygenation burden comparable to those of control subjects. However, within the pSS cohort, impaired pulmonary function—particularly reduced DLCO—was consistently associated with worse nocturnal oxygenation and remained an independent predictor of minimum nocturnal oxygen saturation after adjustment for age, BMI, and OSA severity. Our findings suggest that nocturnal oxygenation in pSS reflects the combined effects of OSA severity and pulmonary dysfunction, particularly impaired diffusion capacity and restrictive ventilatory impairment. Future studies with larger cohorts are warranted to clarify the relative contributions of these physiological mechanisms.

## Figures and Tables

**Figure 1:**
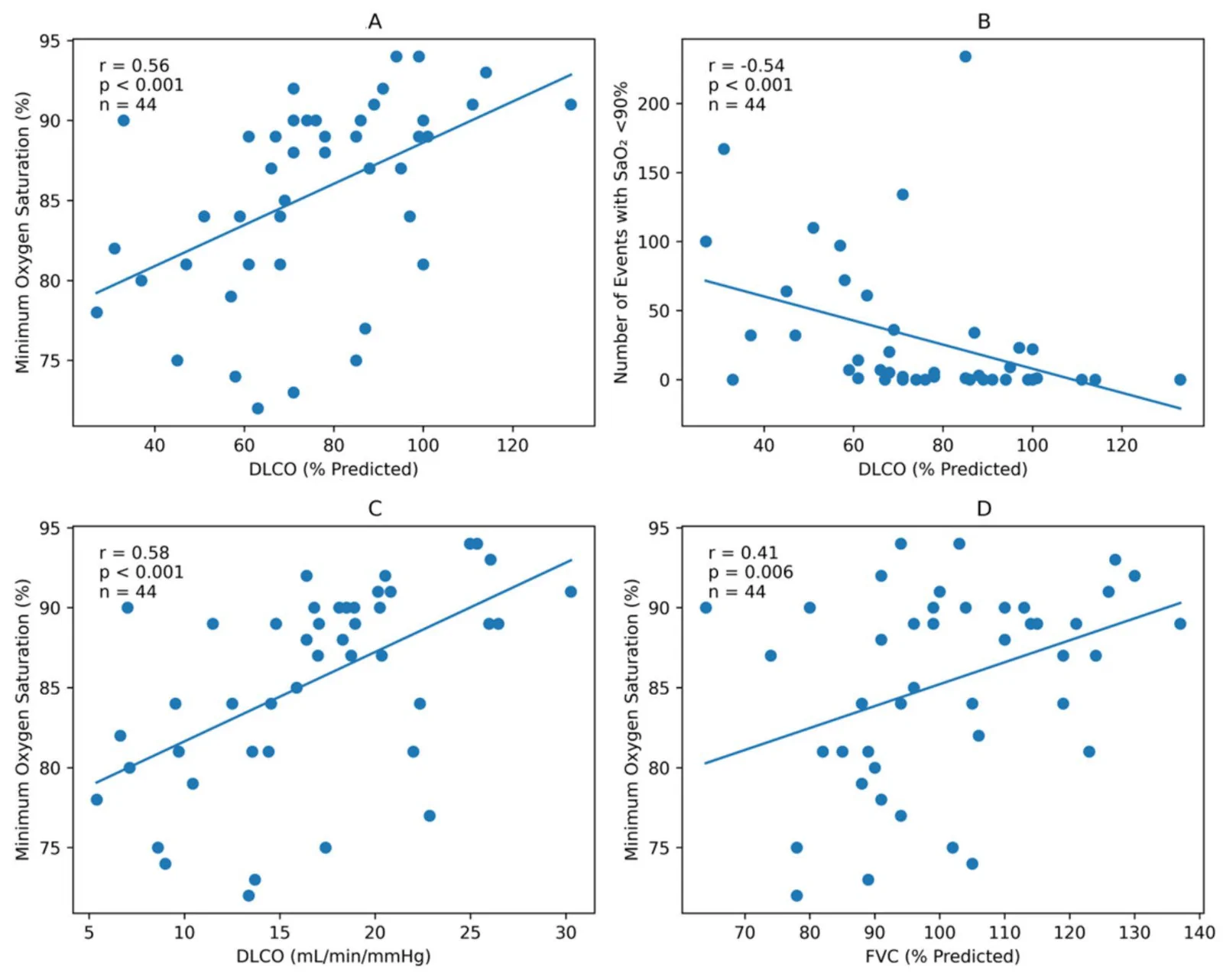
Correlations of DLCO and FVC with nocturnal oxygenation parameters. DLCO expressed as percent predicted was positively correlated with minimum oxygen saturation (**A**) and negatively correlated with the number of events with SaO_2_ < 90% (**B**). Absolute DLCO was positively correlated with minimum oxygen saturation (**C**), and FVC percent predicted was also positively correlated with minimum oxygen saturation (**D**). Each blue dot represents an individual participant, and the blue lines indicate the fitted linear regression lines.

**Table 1:** Baseline Characteristics and Polysomnographic Findings of Patients with Primary Sjögren Syndrome and Controls (*n* = 132).

Variable	PSS (*n* = 44)	Controls (*n* = 88)	*p*-Value
**Demographic and Clinical Characteristics**			
Age, years	56.3 ± 13.2	54.0 ± 8.6	0.172
Female sex, *n* (%)	42 (95.5%)	88 (100.0%)	0.109
BMI, kg/m^2^	29.0 ± 4.6	28.5 ± 4.0	0.422
Neck circumference, cm	**35.0 (33.0–36.2)**	**36.0 (34.0–37.0)**	0.054
Smoking exposure, pack-years	0.0 (0.0–5.2)	0.0 (0.0–15.0)	0.191
Epworth Sleepiness Scale score	5.0 (3.0–7.2)	5.0 (3.0–9.0)	0.579
**Sleep Architecture**			
Stage N3 sleep, %	20.8 ± 11.7	19.3 ± 8.4	0.690
REM sleep, %	**12.0 (6.8–17.2)**	**15.5 (10.8–20.0)**	**0.025**
**Sleep-Disordered Breathing**			
AHI, events/h	12.0 (6.8–21.0)	14.0 (6.8–22.2)	0.822
REM-AHI, events/h	26.0 (12.8–40.0)	24.5 (10.0–38.5)	0.645
NREM-AHI, events/h	11.0 (5.0–18.0)	10.0 (4.0–20.2)	0.950
Supine AHI, events/h	14.0 (7.8–24.5)	18.5 (7.0–36.0)	0.320
Non-supine AHI, events/h	6.0 (2.0–13.0)	6.0 (2.0–15.0)	0.545
**OSA Severity**			
No OSA, *n* (%)	7 (15.9%)	20 (22.7%)	0.360
Mild OSA, *n* (%)	**12 (27.3%)**	**11 (12.5%)**	**0.035**
Moderate OSA, *n* (%)	19 (43.2%)	38 (43.2%)	1.000
Severe OSA, *n* (%)	6 (13.6%)	19 (21.6%)	0.272
**Nocturnal Oxygenation Profile**			
Minimum oxygen saturation, %	87.5 (81.0–90.0)	87.0 (83.0–89.0)	0.940
Oxygen desaturation events, *n*	73.5 (39.8–127.2)	74.0 (39.0–139.5)	0.919
Events with SaO_2_ < 90%, *n*	4.0 (0.0–32.5)	2.5 (1.0–25.0)	0.856
Events with SaO_2_ < 80%, *n*	0.0 (0.0–0.0)	0.0 (0.0–0.0)	0.672
Nocturnal desaturation, *n* (%)	12 (27.3%)	24 (27.3%)	1.000

**Abbreviations:** AHI, apnea–hypopnea index; BMI, body mass index; NREM, non-rapid eye movement sleep; OSA, obstructive sleep apnea; PSS, primary Sjögren syndrome; REM, rapid eye movement sleep; SaO_2_, arterial oxygen saturation.

**Table 2:** Correlations Between Nocturnal Hypoxemia Parameters and Pulmonary Function Variables in Patients with Primary Sjögren Syndrome.

Nocturnal Hypoxemia Parameter	DLCO	DLCO%	DLCO/VA	FVC%
**Minimum SaO_2_ (%)**	r = 0.578, *p* < 0.001	r = 0.556, *p* < 0.001	r = 0.300, *p* = 0.048	r = 0.405, *p* = 0.006
**Desaturation events (*n*)**	r = − 0.349, *p* = 0.020	r = − 0.298, *p* = 0.049	r = − 0.151, *p* = 0.329	r = − 0.085, *p* = 0.585
**Events with SaO_2_ < 90% (*n*)**	r = − 0.576, *p* < 0.001	r = − 0.541, *p* < 0.001	r = − 0.297, *p* = 0.050	r = − 0.378, *p* = 0.011
**Events with SaO_2_ < 80% (*n*)**	r = − 0.289, *p* = 0.057	r = − 0.260, *p* = 0.088	r = − 0.049, *p* = 0.754	r = − 0.260, *p* = 0.089

**Abbreviations:** DLCO, diffusing capacity of the lung for carbon monoxide; DLCO/VA, diffusing capacity corrected for alveolar volume; FVC, forced vital capacity; SaO_2_, arterial oxygen saturation.

**Table 3:** Associates of Minimum Nocturnal Oxygen Saturation in Patients with Primary Sjögren Syndrome.

Variable	β Coefficient	95% CI	*p*-Value
***Univariate***			
Age (years)	−0.247	−0.367 to −0.127	<0.001
BMI (kg/m^2^)	−0.423	−0.808 to −0.037	0.033
Neck circumference (cm)	−0.249	−0.918 to 0.419	0.456
Smoking exposure (pack-years)	−0.067	−0.274 to 0.139	0.515
AHI (events/h)	−0.361	−0.484 to −0.239	<0.001
FVC (% predicted)	0.137	0.034 to 0.240	0.010
FEV1 (% predicted)	0.055	−0.053 to 0.162	0.309
FEV1/FVC (%)	−0.265	−0.527 to −0.002	0.048
DLCO (% predicted)	0.129	0.058 to 0.200	<0.001
DLCO/VA (% predicted)	0.032	−0.072 to 0.135	0.541
VA	2.991	1.515 to 4.467	<0.001
Interstitial involvement (number of segments)	−0.187	−0.468 to 0.093	0.185
Bronchiectatic involvement (number of segments)	−0.176	−0.717 to 0.364	0.513
***Multivariate***			
**Model 1**			
Age (years)	−0.139	−0.248 to −0.029	0.014
BMI (kg/m^2^)	−0.179	−0.472 to 0.115	0.225
AHI (events/h)	−0.270	−0.402 to −0.138	<0.001
**Model 2**			
Age (years)	−0.082	−0.186 to 0.024	0.125
BMI (kg/m^2^)	−0.245	−0.513 to 0.024	0.073
AHI (events/h)	−0.253	−0.373 to −0.134	<0.001
DLCO (% predicted)	0.088	0.032 to 0.144	0.003
**Model 3**			
Age (years)	−0.112	−0.213 to −0.010	0.032
BMI (kg/m^2^)	−0.256	−0.508 to −0.004	0.047
AHI (events/h)	−0.238	−0.350 to −0.125	<0.001
DLCO (% predicted)	0.053	−0.007 to 0.112	0.084
FVC (% predicted)	0.093	0.018 to 0.168	0.017

**Abbreviations:** AHI, apnea–hypopnea index; BMI, body mass index; CI, confidence interval; DLCO, diffusing capacity of the lung for carbon monoxide; DLCO/VA, DLCO corrected for alveolar volume; FEV1, forced expiratory volume in one second; FVC, forced vital capacity; VA, alveolar volume.

**Table 4:** Pulmonary Function and Nocturnal Oxygenation According to Structural Pulmonary Involvement on Thoracic Computed Tomography in Patients with Primary Sjögren Syndrome.

Variable	CT İnvolvement (*n* = 23)	No CT İnvolvement (*n* = 21)	*p*-Value
Minimum SaO_2_, %	82.0 (78.5–88.5)	89.0 (87.0–91.0)	0.002
Desaturation events, *n*	109.0 (56.5–133.5)	61.0 (23.0–113.0)	0.041
Events with SaO_2_ < 90%, *n*	20.0 (2.0–68.0)	0.0 (0.0–5.0)	0.005
Events with SaO_2_ < 80%, *n*	0.0 (0.0–0.5)	0.0 (0.0–0.0)	0.053
AHI, events/h	17.0 (9.5–22.5)	9.0 (6.0–17.0)	0.047
FVC, % predicted	91.0 (88.5–104.5)	110.0 (96.0–124.0)	0.010
DLCO, % predicted	66.0 (52.0–71.0)	91.0 (85.0–99.0)	<0.001

## Data Availability

The original contributions presented in this study are included in the article/Supplementary Material. Further inquiries can be directed to the corresponding author.

## Supplementary Materials

The following supporting information can be downloaded at: https://www.mdpi.com/article/10.3390/biomedicines14102152/s1, Table S1. Polysomnographic parameters according to structural pulmonary involvement in pSS compared with controls.

## Abbreviations

AASM	American Academy of Sleep Medicine
AHI	Apnea–Hypopnea Index
ANA	Antinuclear Antibody
BMI	Body Mass Index
CI	Confidence Interval
CT	Computed Tomography
DLCO	Diffusing Capacity of the Lung for Carbon Monoxide
DLCO/VA	DLCO Corrected for Alveolar Volume
ESS	Epworth Sleepiness Scale
FEV1	Forced Expiratory Volume in One Second
FEV1/FVC	Ratio of Forced Expiratory Volume in One Second to Forced Vital Capacity
FVC	Forced Vital Capacity
ILD	Interstitial Lung Disease
IRB	Institutional Review Board
N3	Stage N3 Sleep/Slow-Wave Sleep
NREM	Non-Rapid Eye Movement
NREM-AHI	Apnea–Hypopnea Index During NREM Sleep
NSIP	Nonspecific Interstitial Pneumonia
ODI	Oxygen Desaturation Index
OSA	Obstructive Sleep Apnea
PSG	Polysomnography
pSS	Primary Sjögren Syndrome
REM	Rapid Eye Movement
REM-AHI	Apnea–Hypopnea Index During REM Sleep
RF	Rheumatoid Factor
SPSS	Statistical Package for the Social Sciences
SSA	Sjögren’s-Syndrome-Related Antigen A/Anti-Ro
SSB	Sjögren’s-Syndrome-Related Antigen B/Anti-La
T90	Time Spent with Oxygen Saturation Below 90%
UIP	Usual Interstitial Pneumonia
VA	Alveolar Volume
