# Supplementary material for: Pulmonary Dysfunction Contributes to Nocturnal Hypoxemia Beyond Obstructive Sleep Apnea Severity in Primary Sjögren Syndrome
Source: Biomedicines. 2026 Sep 23;14(10):2152. doi: 10.3390/biomedicines14102152 (PMC13701980; doi:10.3390/biomedicines14102152)
Supplement: Supplementary file 1 [file biomedicines-14-02152-s001.zip › biomedicines-4550185-supplementary.pdf]

Supplementary Table S1. Polysomnographic parameters according to structural pulmonary involvement in pSS compared with controls

Variable	pSS with structural pulmonary involvement (n=23)	pSS without structural pulmonary involvement (n=21)	Controls (n=88)	p-value: involvement (+) vs controls	p-value: involvement (−) vs controls
REM sleep, %	12.0 (5.5–16.5)	13.0 (10.0–18.0)	15.5 (10.8–20.0)	**0.028**	0.215
N3 sleep, %	20.0 (10.5–29.0)	24.0 (12.0–27.0)	20.5 (13.0–25.0)	0.802	0.366
AHI, events/h	17.0 (9.5–22.5)	9.0 (6.0–17.0)	14.0 (6.8–22.2)	0.346	0.175
REM-AHI, events/h	31.0 (19.0–52.5)	22.0 (6.0–33.0)	24.5 (10.0–38.5)	0.155	0.444
NREM-AHI, events/h	15.0 (7.0–21.5)	7.0 (4.0–16.0)	10.0 (4.0–20.2)	0.234	0.248
Supine AHI, events/h	21.0 (9.0–33.0)	12.0 (7.0–22.0)	18.5 (7.0–36.0)	0.928	0.092
Non-supine AHI, events/h	9.0 (4.5–12.5)	4.0 (2.0–15.0)	6.0 (2.0–15.0)	0.974	0.317
Minimum nocturnal SpO_2, %	82.0 (78.5–88.5)	89.0 (87.0–91.0)	87.0 (83.0–89.0)	**0.033**	**0.017**
Desaturation events, n	109.0 (56.5–133.5)	61.0 (23.0–113.0)	74.0 (39.0–139.5)	0.266	0.180
SpO_2 <90%, events, n	20.0 (2.0–68.0)	0.0 (0.0–5.0)	2.5 (1.0–25.0)	0.053	**0.019**
SpO_2 <80%, events, n	0.0 (0.0–0.5)	0.0 (0.0–0.0)	0.0 (0.0–0.0)	0.124	0.266

Abbreviations: AHI, apnea–hypopnea index; NREM, non-rapid eye movement; pSS, primary Sjögren syndrome; REM, rapid eye movement; SpO_2, peripheral oxygen saturation.
